# Long-Term Nitrogen Addition Drives Contrasting Nutrient Allocation Strategies in Overstory Poplar Trees and Understory Herbs

**DOI:** 10.3390/plants14223548

**Published:** 2025-11-20

**Authors:** Rong Huang, Huihui Liu, Chen Zhang, Yu Fang, Ziqi Shen, Tingting Ren, Honghua Ruan

**Affiliations:** Department of Ecology, Co-Innovation Center for Sustainable Forestry in Southern China, Nanjing Forestry University, Nanjing 210037, China

**Keywords:** N addition, nutrient allocation, poplar plantation, plant organs

## Abstract

Long-term nitrogen (N) addition induces N saturation in forests, but its impacts on nutrient allocation across plant organs and life forms remain unclear. Utilizing a 12-year N addition experiment in a coastal poplar plantation in southeastern China, which included five treatments (0 [CK], 50 [N_1_], 100 [N_2_], 150 [N_3_], and 300 [N_4_] kg N ha^−1^ yr^−1^), we examined N, phosphorus (P), and potassium (K) concentrations in leaves and roots of overstory poplar trees and understory herbs in 2024. Results showed understory leaf N increased by 14.9–31.4% in vigorous growth, while overstory leaf N rose only 7.0% under the highest N treatment. By late growth, N_3_ treatment understory herbs had 17.2% and 21.9% higher leaf and root N, respectively. In contrast, overstory root N decreased by 15.7% under N_1_, but root P increased by 55.4% under N_2_. These findings reveal that understory herbs preferentially allocate nutrients to leaves, whereas overstory poplar trees shift allocation to roots under elevated N. Such divergent strategies may reshape interlayer nutrient competition, thereby affecting forest structure and function and providing theoretical and practical guidance for predicting N addition responses and plantation management.

## 1. Introduction

The concentration of atmospheric reactive nitrogen (N) compounds has been continuously increasing, leading to a significant rise in global N addition rates [1,2]. This atmospheric N addition exerts diverse effects on the nutrient concentration and nutrient cycling processes across ecosystems, impacting soil, leaf, litter, roots, and microorganisms [3,4]. Moderate N addition can enhance plant utilization of soil N in forest ecosystems, thereby alleviating N limitation and promoting plant growth [5]. However, long-term N input may induce soil acidification and nutrient imbalances, which ultimately reduce net primary productivity and impair ecosystem functioning [6]. Furthermore, N addition alters soil transformation and availability, leading to imbalances in the carbon (C)/phosphorus (P) and N/P ratios. This shift may further exacerbate P limitation [7].

N, P and potassium (K) are major nutrients required for plant life activities and play an irreplaceable role in the growth and development process [8]. The allocation patterns of nutrient elements in different plant organs reflect the plant’s ability to acquire, transport, and store nutrients [9,10]. Plant leaves generally have priority in nutrient allocation [11] to ensure normal growth and development. The roots are important organs for nutrient absorption, accumulation, and storage. They play a crucial role in maintaining the nutrient balance of the plant under stress and supporting leaf growth [12]. Currently, many studies focus on the impact of nutrient availability on biomass allocation between leaves and roots [13,14]. In contrast, how nutrients themselves are allocated between the leaves and roots of plants with different life forms, particularly between overstory poplar trees and understory herbs, remains poorly understood.

In order to maximize plant growth and maintain optimal metabolic activity, plants need to balance nutrient allocation between organs under different environmental stresses [15,16]. For example, understory herbs allocate nutrients by adjusting the N/P ratio between leaves, branches, and stems. Leguminous plants allocate more N and P to the branches and stems, while deciduous broadleaf plants with shorter leaf lifespans allocate more nutrients to the leaves [17]. In dry ecosystems, plants increase the nutrient concentration in the leaves to enhance the potential photosynthetic capacity per unit area, achieving higher water use efficiency [18]. Plant nutrient allocation strategies exhibit differences among individual organs. The limited allocation of nutrients in plants, as an important strategy for coping with environmental changes, reflects the influence of plant evolution and ecological processes, as well as trade-offs between various functions [19,20]. Therefore, exploring the differentiation of plant–soil nutrient elements under N addition is crucial for understanding nutrient utilization and allocation in plants of different life forms under N addition, thereby effectively guiding ecosystem management.

We conducted a 12-year N addition experiment in a poplar plantation in the coastal saline land reclamation area of eastern China. Here, our goal is to address the following questions through this study: (1) What are the nutrient allocation strategies between different plant forms (e.g., overstory poplar trees vs. understory herbs) under long-term N application? (2) What are the main soil factors influencing the nutrient allocation in the leaves and roots of different life forms under long-term N application?

We hypothesized that: long-term N addition drives contrasting nutrient allocation strategies between overstory poplar trees and understory herbs due to their distinct life history strategies (conservative vs. opportunistic) and functional trade-offs [21,22]. Understory herbs prioritize allocating the increased N resources to their leaves to maximize photosynthetic rate and competitive growth [23,24], while trees prioritize allocating resources to their roots to alleviate N addition-induced P limitation and enhance their conservative survival ability [1,25].

## 2. Materials and Methods

### 2.1. Study Site and Experimental Design

The research site was located at the Dongtai Forest Farm (32°52′ N, 120°49′ E) in eastern Jiangsu Province, China (Figure S1). This region has a subtropical climate with distinct maritime and monsoon influences. The mean annual temperature (MAT) is 13.7 °C, and the mean annual precipitation (MAP) is 1051 mm [26]. The frost-free period lasts for 220 days. The soil is coastal alluvial saline–alkaline soil, classified as desalinated meadow soil, with a sandy loam texture, and the soil pH is slightly alkaline [27]. The experimental plots were established in 2012, selecting 8-year-old poplar plantations with similar site conditions for N treatments. The understory herbs include: *Causonis japonica*, *Humulus scandens*, *Ophiopogon bodinieri*, *Erigeron annuus*, and *Veronica persica*, among others. As of 2024, the average tree height was 19.38 m, and the average diameter at breast height (DBH) was 30.7 cm.

A randomized block design was used, with four replicated blocks (each 30 m × 190 m, with a spacing of more than 300 m between blocks). Each block was assigned five treatment plots (each plot with an area of 25 m × 30 m, with a 15 m buffer zone between treatments): CK (0 kg N ha^−1^ year^−1^), N_1_ (50 kg N ha^−1^ year ^−1^), N_2_ (100 kg N ha^−1^ year ^−1^), N_3_ (150 kg N ha^−1^ year ^−1^), and N_4_ (300 kg N ha^−1^ year ^−1^) [28,29]. During each growing season (from May to October), ammonium nitrate (NH_4_NO_3_) was applied to the plots. We calculated the total amount of NH_4_NO_3_ required for the year, dissolved one-sixth of it in 20 L of water, and applied it evenly on the surface using a backpack sprayer to simulate natural N addition. In the control plots, an equal volume (20 L) of water was applied.

### 2.2. Plant and Soil Sample Collection

Plant and soil samples were collected during the vigorous growth period (July) and the late growth period (October) in 2024.

Plant sample collection: In each treatment plot, three well-growing and healthy trees were randomly selected. Using a pole pruner, fully expanded, healthy, mature leaves were collected. Ten leaves were collected from each tree, and the leaves from the same treatment were mixed in a resealable bag. Fine root samples were collected from the area around the tree, within a 0.5 m to 1 m radius, using a soil auger with a 7 cm diameter. The samples were then sieved through a 2 mm sieve to isolate poplar fine roots (diameter < 2 mm). In each treatment plot, three 1 m × 1 m subplots were randomly chosen. Using the harvesting method, all leaves and roots of understory herbs within the subplots were collected. All samples were placed in preservation bags and transported back to the laboratory for further processing.

Soil sample collection: In the 20 sample plots, soil samples were collected using a random sampling method. Five sampling points were set up in each plot. The surface litter layer was removed, and a soil auger with a 20 mm diameter was used to collect soil samples from the 0–15 cm soil layer at each of the five points. The soil samples from these five points were then thoroughly mixed together to form a composite sample.

### 2.3. Sample Treatment

The roots and leaves of both overstory poplar trees and understory herbs were separately rinsed with deionized water to remove excess soil and impurities. All plant samples were placed in an oven at 105 °C for 30 min, then dried at 65 °C until a constant weight was achieved. After grinding, the samples were sieved through a 60-mesh screen. P concentration was determined using the molybdenum-antimony anti-absorption photometric method after digestion with H_2_SO_4_-H_2_O_2_ [30].

The collected soil was air-dried after removing roots and gravel, homogenized, and passed through a 2 mm sieve. Soil samples were treated using the alkali fusion method. Soil total P was measured by the molybdenum blue method [31]. Available phosphorus (AP) was extracted using a 0.5 M sodium bicarbonate solution (pH 8.5) and quantitatively analyzed by the molybdenum blue method [31]. The soil ammoniacal N and nitrate N were extracted using a 2 M KCl solution (*w*:*v* = 1:5) and determined via a continuous analytical system (San++, Skalar, Breda, The Netherlands) [32]. Soil moisture concentration (%) was calculated using the formula [(wet weight − dry weight) × 100]/dry weight, where soil dry weight was determined after drying at 105 °C in an oven for 24 h [33]. Soil pH was measured with a glass electrode at a 1:2.5 soil-to-water ratio (METTLER TOLEDO Five Easy Plus FP20) [32]. The total nitrogen (TN) of plant and soil samples was determined using an elemental analyzer (Elementar Vario EL, Langenselbold, Germany). The K concentrations in the digested solutions were determined using an atomic absorption spectrophotometer (Analyst 400, PerkinElmer, Shelton, CT, USA), according to the flame photometry method [34].

### 2.4. Data Analysis

By integrating nutrient concentration data across all plant organs, we employed one-way analysis of variance (ANOVA) coupled with Duncan’s post hoc multiple comparison test to assess the differences in N, P, and K stoichiometric traits of soil, leaves, and fine roots under N addition treatments. Similarly, we used two-way ANOVA to assess the differences between months and treatments on the N, P, and K concentration of different plant organs (Table S1). Tukey’s honest significant difference (HSD) post hoc test was applied to analyze the significant differences among means (*p* < 0.05).

This study used the reduced major axis regression (RMA) method to assess the N, P and K allocation relationship in leaves and fine roots under different N addition treatments. The allocation relationship is described by the formula [35]:

log(Y) = log(β) + α log(X)(1)

where Y and X represent the N, P, and K concentrations in the leaves and roots, respectively, and β and α are the *y*-axis intercept and scale exponent. The difference between α and 1.0 was compared using the R4.4.2 *smatr* package. When the difference between α and 1.0 was significant, it indicated an allometric growth relationship between X and Y; if α > 1.0, it suggests a higher allocation ratio to the leaves, while α < 1.0 indicates a greater allocation to the roots; in other cases, X and Y exhibited isometric growth. Finally, Tukey’s test was used to determine the heterogeneity of α for nutrients under different N addition treatments.

To clarify whether the N, P and K stoichiometric traits of leaves and fine roots in overstory poplar trees and understory herbs are regulated by soil factors, we employed hierarchical partitioning (HP), with plant nutrient concentrations as response variables and soil properties as explanatory variables, to quantify the contribution rates of different soil factors to the variations in these stoichiometric traits [36,37].

All raw data were log-transformed prior to statistical analyses to meet the assumption of normal distribution. The data were processed using Excel 2021, and the experimental data were tested for normality using SPSS 27.0. The data in the figures are presented as means ± standard error. Bar charts and interval plots were created using the R4.4.2 *ggplot2* package, correlation analysis was performed using the R4.4.2 *corrplot* package, and redundancy analysis was performed using the R4.4.2 *vegan* package.

## 3. Results

### 3.1. The Effect of N Addition on Nutrient Content in the Leaves and Roots of Understory Herbs and Overstory Poplar Trees

**Figure 1:**
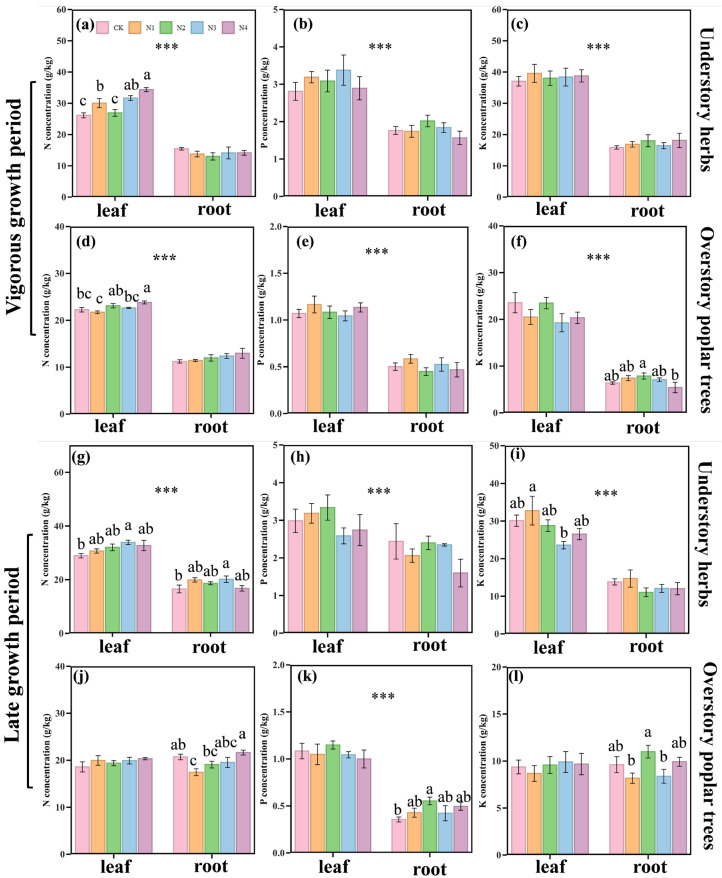
Nitrogen (N) treatment during the vigorous growth period and late growth period of understory herbs and overstory poplar trees, with respect to the concentrations of N, Phosphorus (P), and Potassium (K). *** indicates significant differences in mean values between groups for both leaves and roots *** <0.001); different lowercase letters indicate significant differences in the same organ under different N treatments (*p* < 0.05). (**a**–**c**) N, P, K concentrations in understory herbs (roots/leaves, vigorous growth period); (**d**–**f**) N, P, K concentrations in understory poplar trees (roots/leaves, vigorous growth period); (**g**–**i**) N, P, K concentrations in understory herbs (roots/leaves, late growth period); (**j**–**l**) N, P, K concentrations in understory poplar trees (roots/leaves, late growth period).

Understory herbs leaf N, P and K concentrations were significantly higher than those in the roots during both the vigorous growth period and the late growth period. During the vigorous growth period, the same pattern was observed in overstory poplar trees, consistent with the understory herbs. However, in the late growth period, only the leaf P concentration of overstory poplar trees was significantly higher than that in the roots (Figure 1, *p* < 0.05).

During the vigorous growth period, the leaf N concentration of understory herbs increased significantly with rising N application rates, showing an increase of 14.9% to 31.4% compared to CK (Figure 1a, *p* < 0.05). In contrast, for overstory poplar trees, the leaf N concentration was highest only under the N_4_ treatment, with an increase of 7.0% compared to CK (Figure 1d, *p* < 0.05). In the late growth period, the leaf and root N concentrations of understory herbs in the N_3_ treatment were significantly higher than those in the CK, with increases of 17.2% and 21.9%, respectively (Figure 1g, *p* < 0.05). However, compared to CK, the root N concentration in the N_1_ treatment of overstory poplar trees was significantly lower, showing a decrease of 15.7% (Figure 1j, *p* < 0.05), while the root P concentration in the N_2_ treatment was significantly higher, with an increase of 55.4% (Figure 1k, *p* < 0.05).

### 3.2. The Effect of N Addition on the Scaling Exponents of Nutrients in Leaves and Roots of Understory Herbs and Overstory Poplar Trees

**Figure 2:**
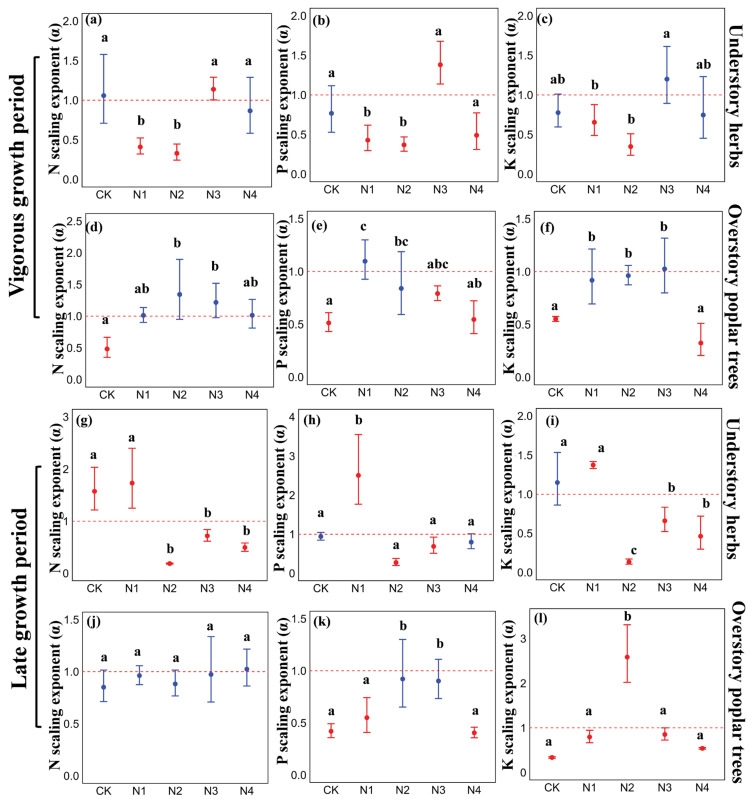
Nutrient scaling indices of understory herbs and overstory poplar trees under N treatment during the vigorous growth period and late growth period, error bars represent the 95% confidence interval of the scaling exponents; the dashed line represents a scaling exponent of 1; different colors indicate whether there is a difference from the scaling exponent of 1—blue indicates no difference from the scaling exponent of 1 (isometric growth), while red indicates a difference from the scaling exponent of 1 (allometric growth), different lowercase letters indicate significant differences in scaling exponents among different N addition treatments (*p* < 0.05). (**a**–**c**) N, P, K scaling exponent in understory herbs (vigorous growth period); (**d**–**f**) N, P, K scaling exponent in understory poplar trees (vigorous growth period); (**g**–**i**) N, P, K scaling exponent in understory herbs (late growth period); (**j**–**l**) N, P, K scaling exponent in understory poplar trees (late growth period).

In the vigorous growth period and late growth period, N addition resulted in significant differences in the nutrient scaling exponent (α) between understory herbs and overstory poplar trees (Tables S3 and S4, Figure 2, *p* < 0.05). During the vigorous growth period, the N, P, and K scaling exponents (α) of understory herbs were significantly less than 1 under the N_1_ and N_2_ treatments (Figure 2a–c, *p* < 0.05), indicating allometric growth with greater resource allocation to the roots. In contrast, those of overstory poplar trees did not differ significantly from 1 under the same treatments (Figure 2d–f), indicating isometric growth.

Compared to the CK, nutrient allocation in understory herbs under the N_1_ and N_2_ treatments shifted towards the roots for N and P during the vigorous growth period (Figure 2a,b, *p* < 0.05). In contrast, overstory poplar trees under the N_2_ treatment showed a tendency to allocate N, P, and K to the leaves (Figure 2d,e, *p* < 0.05). In the late growth period, understory herbs under the N_2_-N_4_ treatments allocated more N and K to the roots compared to the CK, while P allocation under the N_1_ treatment was concentrated in the leaves (Figure 2g–i, *p* < 0.05). For overstory poplar trees, P and K allocation under the N_2_ treatment favored the leaves (Figure 2k,l, *p* < 0.05).

### 3.3. The Effect of Soil Factors on the Content of Nutrients in Leaves and Roots of Understory Herbs and Overstory Poplar Trees

**Figure 3:**
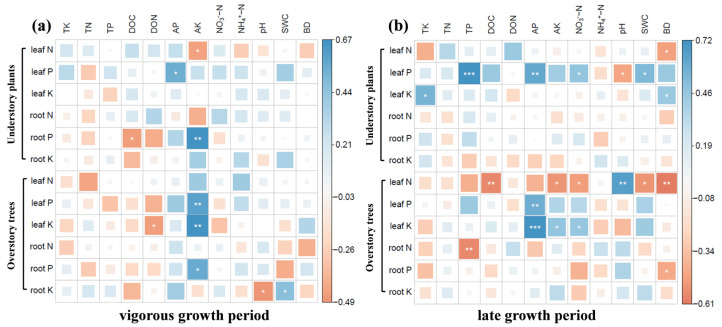
Correlation analysis of plant organ nutrients and soil physicochemical properties under long-term N addition across different growth months (* <0.05, ** <0.01, *** <0.001). (**a**) Correlation between soil factors and plant nutrient contents (vigorous growth period); (**b**) Correlation between soil factors and plant nutrient contents (late growth period).

**Figure 4:**
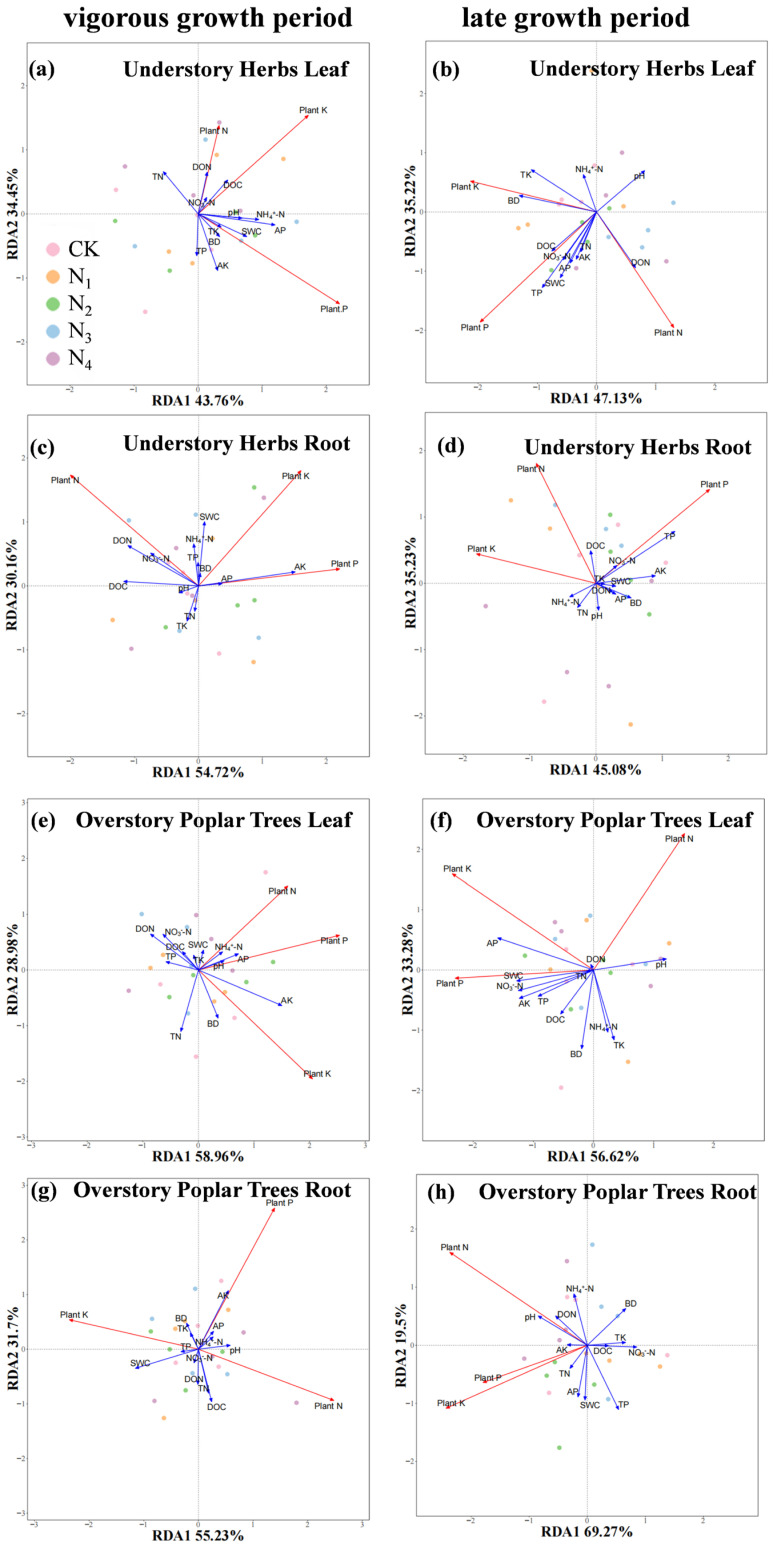
Redundancy analysis (RDA) of leaves, roots, and soil physicochemical properties in overstory poplar trees and understory herbs across different growing seasons under long-term N addition. (**a**,**c**,**e**,**g**) RDA: leaves/roots/soil properties (understory herbs, overstory poplar trees; vigorous growth period); (**b**,**d**,**f**,**h**) RDA: leaves/roots/soil properties (understory herbs, overstory poplar trees; late growth period).

Correlation and redundancy analyses clarified the relationship between the N, P, and K concentration of different plant organs and the soil’s physical–chemical properties (Figure 3 and Figure 4). Combining the changes in nutrient concentration in plant leaves and roots, during the vigorous growth period, the N concentration leaves of understory herbs showed a negative correlation with soil available K (*p* < 0.05), while the N concentration in the leaves of overstory poplar trees showed a positive correlation with soil available K (*p* < 0.05), with the influence of other soil factors not reaching a significant level. Results from HP indicated that soil available K during the vigorous growth period had an importance value of 11.3–27.0% for nutrient concentration in different plant organs (Figure S2a,c,e,g), reaching significant levels for the roots of understory herbs and the leaves and roots of overstory poplar trees. This suggests that soil available K was a key driving factor affecting the N concentration and nutrient allocation in the leaves of understory herbs and overstory poplar trees. In the late growth period, soil bulk density (BD) and total potassium (TK) significantly influenced the N concentration in the leaves of understory herbs and the N and P concentration in the roots of overstory poplar trees (*p* < 0.05), making them the main driving factors for the N concentration in the leaves of understory herbs and the N and P concentration in the roots of overstory trees. The importance values of TK, TP, AP, and SWC for nutrient concentrations in different plant organs reached their maximums, at 24.4%, 30.1%, 26.9%, and 21.8%, respectively (Figure S2b,d,f,h). This indicates that the nutrient concentrations of understory herbs and overstory poplar trees were influenced by multiple soil factors during the late growth period.

## 4. Discussion

### 4.1. The Effect of N Addition on the N, P, and K Content in the Leaves and Roots of Different Plants

Our results indicate that there was a significant difference in the nutrient concentrations of leaves and roots between overstory poplar trees and understory herbs in response to long-term N addition. This may be attributed to differences in their root structures and nutrient absorption characteristics [38]. Additionally, the leaf N concentration of understory herbs increased significantly with rising N application rates during the vigorous growth period (Figure 1a). This finding aligns with previous studies and can be attributed to the co-limitation of light and N for understory herbs [39,40,41]. While N addition alleviates N limitation, light remains the primary constraint. Consequently, the enhanced N availability allows for the synthesis of more photosynthetic enzymes [42]; however, the persistent light limitation prevents the full utilization of these resources for growth, leading to an accumulation of N in the leaves [43].

In contrast, the leaf N concentration of overstory poplar trees exhibited a significant increase only under the highest N treatment (N_4_, Figure 1d). This suggests that overstory poplar trees, which are not light-limited, initially utilize the added N to fuel growth and photosynthesis, which are primarily constrained by soil N availability [44,45]. Under low to moderate N additions (N_1_–N_3_), this growth response likely dilutes the leaf N pool, resulting in no significant change in concentration [7]. Only when N supply exceeds the immediate growth demands—reaching an absorption threshold under the N_4_ treatment—does excess N accumulate in the leaves [46].

Furthermore, our analysis of nutrient scaling exponents revealed distinct allocation strategies. Under the N_2_ treatment, understory herbs preferentially allocated N and P to their roots (α < 1, Figure 2a,b), whereas overstory poplar trees allocated P and K to their leaves (α > 1 or not significantly different from 1, Figure 2e,f,k,l). This divergence supports our hypothesis of contrasting life-history strategies. For understory herbs, once N limitation is partially relieved, investing in root growth is a strategic response to more effectively acquire other potentially limiting resources, such as water and mineral nutrients, from the soil [47,48]. Strengthening the root system enhances their capacity to capture a wider range of resources, thereby maximizing overall fitness in a competitive environment [49].

Conversely, for overstory poplar trees under sufficient N supply (N_2_), the critical limitation may shift to other elements. Allocating limited P and K resources to the leaves, the primary sites of photosynthesis, can enhance photosynthetic efficiency and support overall C gain and growth [50,51,52]. The fact that this pattern was most evident under the N_2_ treatment is ecologically significant. The N_1_ treatment may have been insufficient to fully alleviate N limitation in this ecosystem [44], while the higher N_3_ and N_4_ treatments might have induced secondary stresses, such as soil acidification, ion imbalances, or ammonium/nitrate toxicity, which can disrupt normal nutrient uptake and allocation patterns [53,54].

### 4.2. The Impact of Soil Factors on Nutrient Elements in Different Plant Organs

It is well-established that soil and environmental factors collectively shape plant ecological stoichiometry [55,56]. The results showed that soil TK concentration did not change significantly across different growth months, while soil available K concentration during the vigorous growth period was significantly lower than that in the late growth period (Table S2). This is because plants have a much higher demand for K absorption during the vigorous growth period compared to the late growth period, and the conversion rate of soil non-exchangeable K to available K cannot compensate for the consumption caused by the rapid K uptake of plants. In contrast, the plant’s demand for K absorption weakens in the late growth period, allowing soil K to accumulate [57]. Our study identifies soil available K as a key driver of leaf N concentration and nutrient partitioning in both plant forms during the vigorous growth period (Figure 3 and Figure 4a,e). This period is characterized by high demand for efficient photosynthesis and rapid growth. Potassium plays a fundamental role in these processes, including enzyme activation, stomatal regulation, and photosynthate translocation [58,59].

The negative correlation between soil available K and leaf N in understory herbs suggests that K insufficiency may impair N utilization efficiency, leading to N accumulation in leaves without proportional growth benefits. Conversely, the positive correlation in overstory poplar trees implies that adequate K availability facilitates effective N use and transport to the leaves, supporting their high metabolic activity. Thus, the availability of K appears to modulate the efficiency of N utilization and the subsequent partitioning patterns between leaves and roots in both life forms. This provides critical empirical evidence for a synergistic “K–N” mechanism governing plant growth and nutrient allocation, underscoring that nutrient interactions, rather than N availability alone, are crucial for understanding ecosystem responses to N addition.

## 5. Conclusions

This study investigated the effects of long-term N application on the concentrations of N, P and K in the leaves and roots of overstory poplar trees and understory herbs. The results showed that during the vigorous growth period, the concentrations of N, P, and K in the leaves of both overstory poplar trees and understory herbs were higher than those in the roots. In the late growth period, however, only the leaf P concentration of overstory poplar trees was significantly higher than that in the roots, while there were no significant differences in N and K concentrations between leaves and roots. Notably, with the increase in N application rate, the leaf N concentrations of understory herbs and overstory poplar trees increased by 31.4% and 7.0%, respectively, under the N_4_ treatment during the vigorous growth period. In the late growth period, the leaf and root N concentrations of understory herbs showed an upward trend under the N_3_ treatment, increasing by 17.2% and 21.9%, respectively, while the root N concentration of overstory poplar trees significantly decreased by 15.7% under the N_1_ treatment.

Plants exhibited distinct nutrient allocation preferences under long-term N application: understory herbs allocated more nutrients to roots, whereas overstory poplar trees predominantly allocated nutrients to leaves. The nutrient concentrations of different plant organs were influenced by various soil factors. During the vigorous growth period, soil available K had a more significant impact on the N, P, and K concentrations of plants. The results indicated that the leaves and roots of overstory poplar trees and understory herbs adopted differentiated nutrient allocation strategies under long-term N application. These findings deepen our understanding of the dynamic changes in plant N, P, and K under long-term N input, suggesting that future research should focus on the interaction mechanisms between soil, microorganisms, and plants to further clarify the driving factors behind plant nutrient allocation.

## Data Availability

Data are contained within the article and Supplementary Materials.

## Supplementary Materials

The following supporting information can be downloaded at: https://www.mdpi.com/article/10.3390/plants14223548/s1, Figure S1: (a) Map of the study area showing the location of the poplar plantation site in southeastern China. (b) Four replicated blocks of poplar plantations were established using a randomized block design, with five N addition treatments, which included CK, N_1_, N_2_, N_3_, N_4_ (i.e., 0, 50, 100, 150, and 300 kg N ha^−1^ yr^−1^), respectively; Figure S2: Analysis of the relative importance of soil factors on N, P, and K nutrients in different plant organs across growth seasons under long-term nitrogen addition, using Hierarchical Partitioning (HP). The orange and blue colors represent significant and non-significant results, respectively. Note: TK (Total Potassium), TN (Total Nitrogen), TP (Total Phosphorus), DOC (Dissolved Organic Carbon), DON (Dissolved Organic Nitrogen), AP (Available Phosphorus), AK (Available Potassium), NO_3_^-^-N (Ammonium Nitrogen), NH_4_^+^-N (Nitrate Nitrogen), SWC (Soil Water Content), BD (Bulk Density); Table S1: Effects of long-term N addition: ANOVA of nutrient contents across plant organs; Table S2: Soil physicochemical properties under different treatments in a coastal poplar plantation during the vigorous growth period and late growth period; Table S3: Reduced Major Axis (RMA) regression of the nutrient relationships between leaves and roots of overstory poplar trees and understory herbs under different N addition treatments during the vigorous growth period; Table S4: Reduced Major Axis (RMA) regression of the nutrient relationships between leaves and roots of overstory poplar trees and understory herbs under different N addition treatments during the late growth period.
