# Supplementary material for: Long-Term Nitrogen Addition Drives Contrasting Nutrient Allocation Strategies in Overstory Poplar Trees and Understory Herbs
Source: Plants (Basel). 2025 Nov 20;14(22):3548. doi: 10.3390/plants14223548 (PMC12656388; doi:10.3390/plants14223548)
Supplement: Supplementary file 1 [file plants-14-03548-s001.zip › plants-3962412-supplementary.pdf]

Supplementary Materials:

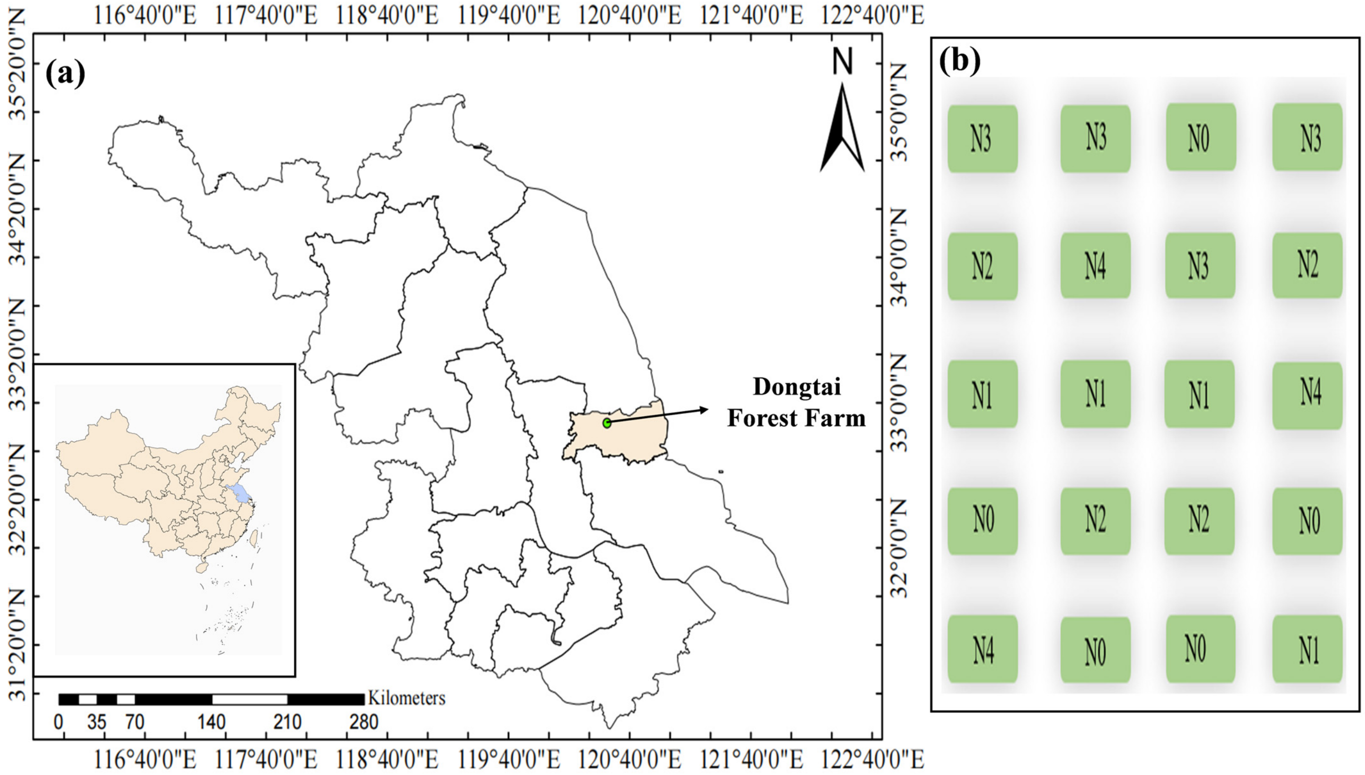

Figure S1. (a) Map of the study area showing the location of the poplar plantation site in southeastern China. (b) Four replicated blocks of poplar plantations were established using a randomized block design, with five N addition treatments, which included CK, N_1, N_2, N_3, N_4 (i.e., 0, 50, 100, 150, and 300 kg N $ha^{-1}yr^{-1}$), respectively.

Table S1. Effects of long-term N addition: ANOVA of nutrient contents across plant organs.

		Overstory Poplar Trees leaf		Overstory Poplar Trees root		Understory herbs leaf		Understory herbs root	
	Factors	*F*	*p*	*F*	*p*	*F*	*p*	*F*	*p*
	Months	72.4559	<0.001	314.8304	<0.001	6.7362	<0.05	38.6492	<0.001
	Treatment	2.3162	0.08291	4.4775	<0.01	9.9227	<0.001	0.8369	0.51261
N	Months*Treatment	1.0744	0.38855	2.0129	0.120997	2.5838	0.05961	2.2349	0.08886
	Months	0.875	0.3579	2.6239	0.11689	0.5112	0.4808	8.2337	<0.01
	Treatment	0.503	0.7338	0.6689	0.61916	1.171	0.3456	2.7554	<0.05
P	Months*Treatment	1.104	0.3749	2.3054	0.08403	1.6525	0.1901	0.6411	0.637799
	Months	308.5202	<0.001	37.6046	<0.001	49.6093	<0.001	21.9616	<0.001
	Treatment	1.6686	0.1863	2.4794	0.06515	1.3998	0.2579	0.3204	0.8618
K	Months*Treatment	1.8517	0.1481	2.6344	0.05299	1.193	0.3341	1.1689	0.3465

Table S2. Soil physicochemical properties under different treatments in a coastal poplar plantation during the vigorous growth period and late growth period.

months	treatment	TK (g/kg)	TN (g/kg)	TP (g/kg)	DOC (mg/kg)	DON (mg/kg)	AP (mg/kg)	AK (mg/kg)	NO_3^--N (mg/kg)	NH_4^+-N (mg/kg)	pH	SWC (%)	BD (g/m3)
Vigorous growth period		17.42±	1.37±	0.68±	43.40±	27.95±	11.66±	86.83±	7.62±	1.03±	8.16±	28.28±	1.29±
	CK	0.16 b	0.10 ab	0.04	3.88	1.07 b	2.80	9.32 a	0.65 b	0.11	0.02	0.37	0.04
		18.69±	1.55±	0.65±	44.07±	33.28±	20.41±	67.75±	9.78±	1.17±	8.14±	28.36±	1.27±
	N_1	0.41 a	0.04 a	0.02	5.66	4.05 b	3.38	4.31 ab	1.66 b	0.24	0.01	0.62	0.02
		17.58±	1.58±	0.69±	36.50±	26.37±	17.77±	75.55±	8.21±	0.86±	8.16±	28.98±	1.25±
	N_2	0.27 b	0.13 a	0.01	1.47	1.54 b	3.60	7.63 ab	0.82 b	0.16	0.05	0.85	0.04
		17.77±	1.21±	0.72±	42.70±	34.50±	18.93±	59.95±	9.99±	0.85±	8.15±	27.75±	1.20±
	N_3	0.12 b	0.06 b	0.03	7.30	8.51 b	1.88	4.11 b	2.01 b	0.20	0.04	0.70	0.03
		17.63±	1.59±	0.69±	44.67±	56.57±	17.32±	71.33±	22.04±	1.02±	8.12±	28.19±	1.23±
	N_4	0.21b	0.15 a	0.02	1.46	12.37 a	2.02	8.18 ab	6.30 a	0.12	0.02	0.68	0.01
Late growth period		18.48±	1.33±	0.70±	51.48±	16.49±	14.99±	110.39±	14.97±	2.82±	7.70±	31.47±	1.29±
	CK	0.78 a	0.10 ab	0.02	7.04	2.58 b	1.04	6.34 a	3.06	2.13	0.10	0.56	0.04
		17.65±	1.24±	0.72±	51.41±	14.46±	15.98±	78.59±	13.36±	0.93±	7.69±	29.63±	1.27±
	N_1	0.41 ab	0.12 b	0.03	10.88	1.13 b	0.51	3.56 b	2.85	0.12	0.10	0.77	0.02
		16.62±	1.36±	0.73±	48.55±	20.67±	20.14±	107.71±	10.74±	0.39±	7.69±	31.52±	1.25±
	N_2	0.31 bc	0.18 ab	0.04	12.56	3.52 b	2.58	10.64 a	2.53	0.03	0.08	1.10	0.04
		15.44±	1.27±	0.70±	54.42±	23.77±	17.04±	102.06±	14.43±	0.76±	7.69±	31.11±	1.20±
	N_3	0.23 cd	0.11 ab	0.03	8.41	1.85 b	2.72	12.18 ab	2.17	0.16	0.09	0.85	0.03
		14.96±	1.66±	0.65±	60.89±	33.98±	19.28±	96.74±	14.31±	1.03±	7.62±	31.29±	1.23±
	N_4	0.48 d	0.08 a	0.05	6.14	4.76 a	3.02	5.20 ab	2.00	0.42	0.06	1.04	0.01

Note: TK (Total Potassium), TN (Total Nitrogen), TP (Total Phosphorus), DOC (Dissolved Organic Carbon), DON (Dissolved Organic Nitrogen), AP (Available Phosphorus), AK (Available Potassium), NO_3^--N (Ammonium Nitrogen), NH_4^+-N (Nitrate Nitrogen), SWC (Soil Water Content), BD (Bulk Density); CK represents the treatment without fertilizer. N1, N2, N3, and N4 represent the nitrogen addition doses of 50, 100, 150, and 300 kg ha^{-1} a^{-1}, respectively; different lowercase letters indicate significant differences among the N treatments.

Table S3. Reduced Major Axis (RMA) regression of the nutrient relationships between leaves and roots of overstory poplar trees and understory herbs under different N addition treatments during the vigorous growth period.

		Overstory Poplar Trees				Understory Herbs			
	treatment	α	95%CI	R^2	*p*	α	95%CI	R^2	*p*
N	CK	**0.48 a**	**(0.35,0.67)**	**0.78**	**0.000**	1.06 a	(0.71,1.58)	0.66	0.766
	N_1	1.01 ab	(0.90,1.14)	0.97	0.821	**0.41 b**	**(0.32,0.52)**	**0.88**	**0.000**
	N_2	1.34 b	(0.95,1.90)	0.75	0.088	**0.33 b**	**(0.24,0.45)**	**0.81**	**0.000**
	N_3	1.22 b	(0.98,1.52)	0.90	0.077	**1.14 a**	**(1.00,1.29)**	**0.97**	**0.044**
	N_4	1.01 ab	(0.81,1.26)	0.90	0.896	0.87 a	(0.58,1.29)	0.66	0.449
P	CK	**0.51 a**	**(0.43,0.61)**	**0.94**	**0.000**	0.77 a	(0.53,1.12)	0.70	0.151
	N_1	1.10 c	(0.93,1.30)	0.94	0.252	**0.43 b**	**(0.30,0.62)**	**0.72**	**0.000**
	N_2	0.84 bc	(0.59,1.19)	0.75	0.295	**0.37 b**	**(0.29,0.47)**	**0.88**	**0.000**
	N_3	**0.79 abc**	**(0.72,0.86)**	**0.98**	**0.000**	**1.38 a**	**(1.14,1.68)**	**0.92**	**0.004**
	N_4	**0.55 ab**	**(0.41,0.72)**	**0.84**	**0.001**	**0.49 a**	**(0.31,0.77)**	**0.55**	**0.004**
K	CK	**0.55 a**	**(0.53,0.58)**	**1.00**	**0.000**	**0.78 ab**	**(0.60,1.01)**	**0.86**	**0.059**
	N_1	0.92 b	(0.69,1.21)	0.84	0.518	**0.65 b**	**(0.49,0.88)**	**0.82**	**0.008**
	N_2	0.96 b	(0.87,1.06)	0.98	0.393	0.35 b	(0.24,0.51)	0.69	0.000
	N_3	1.03 b	(0.80,1.32)	0.87	0.825	1.20 a	(0.89,1.61)	0.82	0.203
	N_4	**0.32 a**	**(0.21,0.51)**	**0.55**	**0.000**	0.75 ab	(0.45,1.23)	0.45	0.234

Note: Bold font indicates that the slope is significantly different from 1. CK represents the control treatment without fertilizer. N_1, N_2, N_3, and N_4 correspond to N addition doses of 50, 100, 150, and 300 $kg \cdot ha^{-2} \cdot a^{-1}$, respectively. Different lowercase letters indicate significant differences ($P < 0.05$) in α values among nitrogen addition treatments. CI stands for confidence interval.

Table S4. Reduced Major Axis (RMA) regression of the nutrient relationships between leaves and roots of overstory poplar trees and understory herbs under different N addition treatments during the late growth period.

		Overstory Poplar Trees				Understory Herbs			
	treat-ment	α	95%CI	R^2	*p*	α	95%CI	R^2	*p*
N	CK	0.85 a	(0.71,1.02)	0.94	0.069	**1.57 a**	**(1.22,2.04)**	**0.86**	**0.002**
	N_1	0.96 a	(0.88,1.06)	0.98	0.384	**1.73 a**	**(1.25,2.40)**	**0.78**	**0.003**
	N_2	0.88 a	(0.77,1.02)	0.96	0.075	**0.19 b**	**(0.18,0.20)**	**0.99**	**0.000**
	N_3	0.97 a	(0.71,1.34)	0.79	0.855	**0.72 b**	**(0.62,0.84)**	**0.95**	**0.001**
	N_4	1.02 a	(0.86,1.22)	0.94	0.767	**0.50 b**	**(0.42,0.58)**	**0.95**	**0.000**
P	CK	**0.42 a**	**(0.36,0.49)**	**0.95**	**0.000**	0.94 a	(0.85,1.04)	0.98	0.216
	N_1	**0.55 a**	**(0.41,0.74)**	**0.81**	**0.001**	**2.51 b**	**(1.77,3.55)**	**0.75**	**0.000**
	N_2	0.92 b	0.65,1.30)	0.75	0.615	**0.28 a**	**(0.20,0.38)**	**0.78**	**0.000**
	N_3	0.90 b	(0.73,1.11)	0.91	0.296	**0.69 a**	**(0.51,0.93)**	**0.82**	**0.018**
	N_4	**0.40 a**	**(0.36,0.46)**	**0.97**	**0.000**	0.80 a	(0.63,1.01)	0.88	0.060
K	CK	**0.34 a**	**(0.32,0.35)**	**1.00**	**0.000**	1.15 a	(0.86,1.53)	0.83	0.310
	N_1	**0.79 a**	**(0.67,0.95)**	**0.94**	**0.015**	**1.37 a**	**(1.33,1.42)**	**1.00**	**0.000**
	N_2	**2.58 b**	**(2.02,3.31)**	**0.87**	**0.000**	**0.13 c**	**(0.11,0.17)**	**0.88**	**0.000**
	N_3	**0.85 a**	**(0.73,1.00)**	**0.95**	**0.049**	**0.66 b**	**(0.52,0.83)**	**0.89**	**0.002**
	N_4	**0.54 a**	**(0.52,0.56)**	**1.00**	**0.000**	**0.46 b**	**(0.30,0.72)**	**0.58**	**0.002**

Note: Bold font indicates that the slope is significantly different from 1. CK represents the control treatment without fertilizer. N_1, N_2, N_3, and N_4 correspond to N addition doses of 50, 100, 150, and 300 kg·ha^{-2}·a^{-1}, respectively. Different lowercase letters indicate significant differences ($P < 0.05$) in α values among nitrogen addition treatments. CI stands for confidence interval.

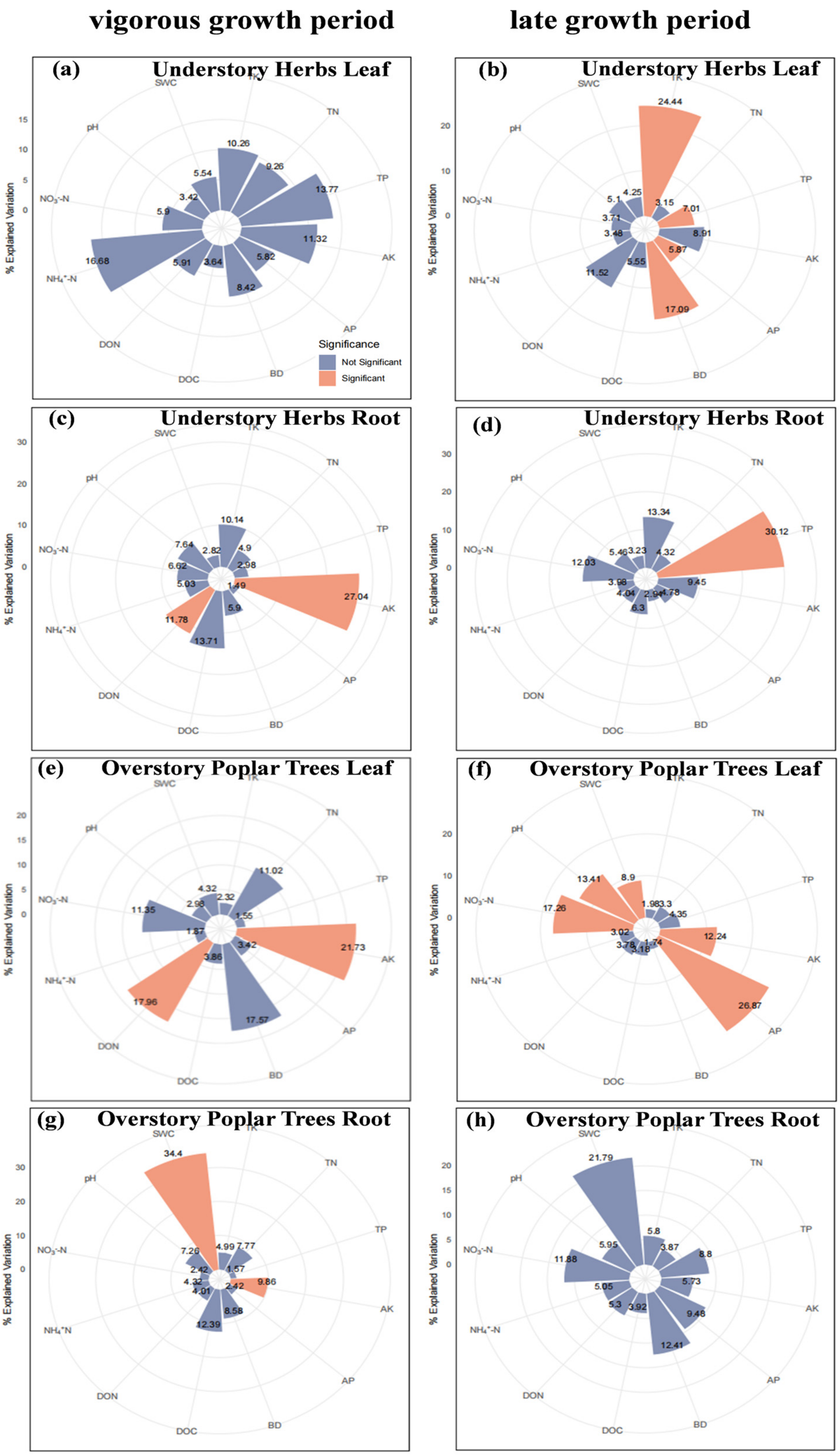

Figure S2. Analysis of the relative importance of soil factors on N, P, and K nutrients in different plant organs across growth seasons under long-term nitrogen addition, using Hierarchical Partitioning (HP). The orange and blue colors represent significant and non-significant results, respectively. Note: TK (Total Potassium), TN (Total Nitrogen), TP (Total Phosphorus), DOC (Dissolved Organic Carbon), DON (Dissolved Organic Nitrogen), AP (Available Phosphorus), AK (Available Potassium), NO_3^--N (Ammonium Nitrogen), NH_4^+-N (Nitrate Nitrogen), SWC (Soil Water Content), BD (Bulk Density).
